# An audit on consent to treatment within forensic inpatient units at the Newsam Centre

**DOI:** 10.1192/bjo.2021.83

**Published:** 2021-06-18

**Authors:** Elisabeth Bond, Stephanie Vel En Tial, Clare Stephenson

**Affiliations:** Leeds and York Partnership NHS Trust

## Abstract

**Aims:**

We aimed to investigate the adherence to the Mental Health Act Code of Practice and the adequate documentation of consent to treatment across three forensic low secure inpatient units.

**Method:**

Our sample included all inpatients detained on three forensic wards at The Newsam Centre. This included a total of 31 patients with an age range of 25 to 59 years. The Mental Health Act Code of Practice was used as criteria for audit standards. Data were collected using Microsoft Excel and analysed using descriptive methods.

**Result:**

We found that 28 patients out of 31 had been admitted for over three months and of these patients 12 were subject to a T2 and 16 subject to a T3. A total of 24 patients had their CTT medication list documented on the online drug chart; with a remaining seven patients who did not. As per guidelines, 27 patients had the appropriate medications prescribed as per their CTT, however one patient did not. The audit revealed a total of two patients currently on a Section 62. Of the qualifying T3 forms, four patients had this reviewed every two years whilst there was one patient who had not.

**Conclusion:**

We found that the adherence to Mental Health Act Code of Practice was overall positive with the majority of service users being reviewed appropriately and documented as per guidance. However, areas identified for improvement included the recording of CTT on online drug charts as well as reviewing T3 every two years. This audit highlights the need for easy access to guidance, appropriate documentation as well as frequent checking of adherence. A leaflet has been created outlining the guidelines and will be distributed to all staff working within the forensic settings and placed in easily accessible locations. As further recommendations from this audit we advise all wards to plan weekly checks during team meetings to ensure information is up to date and that all staff are aware of any discrepencies. A re-audit is planned in the coming months to re-assess adherance after implementation of the interventions.

